# Microbial Competition in Polar Soils: A Review of an Understudied but Potentially Important Control on Productivity

**DOI:** 10.3390/biology2020533

**Published:** 2013-03-27

**Authors:** Terrence H. Bell, Katrina L. Callender, Lyle G. Whyte, Charles W. Greer

**Affiliations:** 1 Department of Natural Resource Sciences, McGill University, Sainte-Anne-de-Bellevue, Quebec H9X 3V9, Canada; E-Mails: terrence.bell@umontreal.ca (T.H.B.); katrina.callender@cnrc-nrc.gc.ca (K.L.C.); lyle.whyte@mcgill.ca (L.G.W.); 2 National Research Council Canada, Energy, Mining and Environment, 6100 Royalmount Avenue, Montreal, Quebec H4P 2R2, Canada

**Keywords:** competition, Arctic, Antarctic, bacteria, fungi, biogeochemistry, biodegradation, soil, microbial communities

## Abstract

Intermicrobial competition is known to occur in many natural environments, and can result from direct conflict between organisms, or from differential rates of growth, colonization, and/or nutrient acquisition. It has been difficult to extensively examine intermicrobial competition *in situ*, but these interactions may play an important role in the regulation of the many biogeochemical processes that are tied to microbial communities in polar soils. A greater understanding of how competition influences productivity will improve projections of gas and nutrient flux as the poles warm, may provide biotechnological opportunities for increasing the degradation of contaminants in polar soil, and will help to predict changes in communities of higher organisms, such as plants.

## 1. Introduction

Although many ecosystem processes are dependent on the growth and activity of multiple species, the productivity of particular individuals can often be limited by the presence of competitors. The classic ecological example of Connell’s barnacles [1] demonstrates that the area potentially occupied by a particular species (*Chthamalus stellatus*), can be greater than its true distribution in the presence of a competitor (*Balanus balanoides*). Such relationships promote biodiversity in many environments, as they prevent complete dominance by the small number of organisms that are best adapted to quickly processing limiting nutrients [2,3,4]. In such cases, competition can constrain specific functions of a community, as the survival and activity of certain organisms limits the resources and habitat available to the most productive species.

When considering the microbial world, productivity can be defined as the rate and efficiency with which any target metabolic function occurs. Growth and accumulation of biomass are easy to picture as productive processes, but the degradation of substrates or the cycling of nutrients can also be considered productive from a microbial perspective (e.g., allowing increased activity or growth), and sometimes from a human perspective (e.g., reduction of environmental contaminants). Although microbial productivity is a universally important component of biogeochemical cycling across environments, the factors that control productivity are especially interesting in polar soils.

Firstly, climate warming and other human disturbances are exposing formerly frozen landscapes to increased temperatures, which will likely lead to more rapid cycling of stored organic material and nutrients. Even small amounts of warming can have large effects on microbial community structure and function in polar soils [5,6], which will inevitably shift the competitive dynamic between taxa. On the other hand, the short Arctic summer limits the highly active period for many microorganisms. For human applications, such as the use of native microbial populations in bioremediation, this means maximizing microbial activity over a short period of time. The exploitation of intermicrobial competition has previously been explored for applied purposes such as the treatment of pathogens (e.g., [7,8]), optimization of agriculture (e.g., [9,10]), and food preservation [11], and has recently been investigated as a means to optimize bioremediation in the Arctic [12].

**Figure 1:**
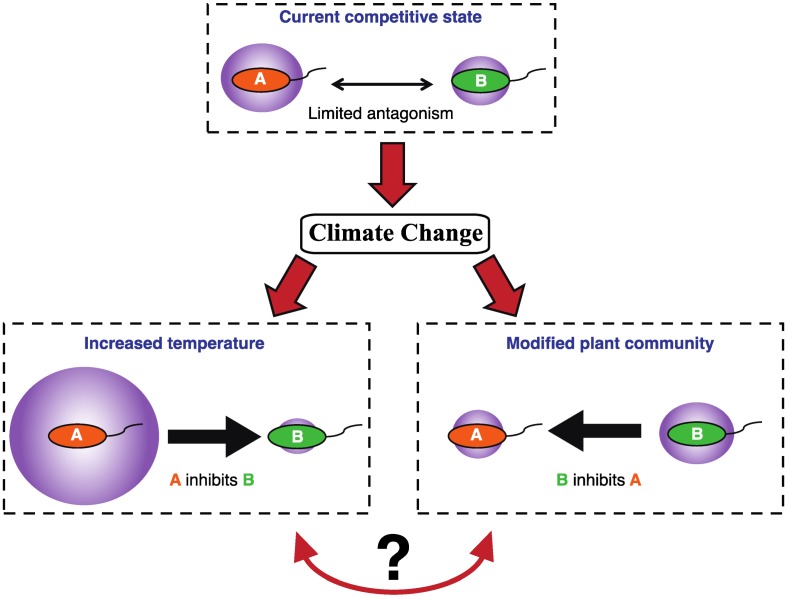
Large environmental shifts such as climate change will alter many aspects of polar soil environments that will shift the growth and activity of microbial species. Although some changes may benefit multiple species in isolation, changes in competitive interactions may determine the ultimate productivity of the whole community. In this scenario, climate change causes changes in both temperature and plant communities. Species A is promoted disproportionately by temperature and suppresses species B, leading to higher productivity (purple circle) by species A, and thus by the overall community. Species B gains a competitive advantage in the new plant community, and suppresses species A, but is not as productive as species A, leading to a decline in overall productivity. It is mostly unknown which factors will be the most important in determining competitive outcomes following climate change, and thus changes in productivity are difficult to predict.

This review will highlight the factors that are known to influence microbial abundance and community structure in polar soils, and how these shifts affect important functions that are mediated by microbial communities. Very few studies have explicitly shown how microbial competition affects function in soils (fewer still in polar regions), but we will attempt to point out areas in which competition may play an important role in limiting or promoting the activity of specific microbial functions. While future warming will likely lead to more active microbial populations, it may also shift the competitive dynamic between microorganisms (Figure 1). Understanding how competition affects key microbial processes will improve predictions of future gas and nutrient fluxes, and may open important biotechnological opportunities.

### 1.1. Microbial Diversity and Productivity

Studies on the relationship between biodiversity and productivity have been performed in many areas of ecology, but have yielded inconsistent results (e.g., [13,14,15]). The addition of species should be expected to increase the productivity of specific functions when species niches are complementary. This may not be the case when the activity of certain key organisms is limited by a lack of resources and space, or by direct inhibition from competitors. An analysis of 180 two-species bacterial cultures showed that almost all pairings resulted in competitive relationships that reduced CO_2_ production relative to monocultures of each species [16]. In multi-species communities, the presence or absence of specific key phylotypes appears to be more important than the overall number of microbial strains in determining productivity in some cases [17,18,19]. Reducing diversity and/or microbial biomass has even been shown to lead to higher productivity with respect to certain functions such as decomposition, nutrient uptake, and bioremediation [12,20,21,22].

### 1.2. Microbial Competition in Polar Soils

Several reviews have highlighted the extent and importance of intermicrobial competition in natural environments [23,24], but few studies have characterized competition in polar environments, with only a handful examining competition among polar soil microorganisms (Table 1). A single gram of soil may contain thousands of microbial species [25] as well as a complex network of interactions. Despite the fact that many polar soils frequently experience extreme cold temperatures, low water content, and intermittently available nutrients, recent molecular studies have shown that the microbial diversity and community composition in these regions resembles what has been observed at lower latitudes [26,27]. Interspecies relationships will also be dynamic, as the growing portion of an Arctic soil community has been shown to vary substantially throughout the year [28].

**Table 1:** Studies that have examined intermicrobial competition in polar soils.

Habitat	Antagonists	Function(s) affected	Proposed mechanism(s) of competition	* Special notes	Reference
*In vitro*					
Moss-covered and barren soil in Svalbard, Norway	*Actinobacteria (Arthrobacter), Gammaproteobacteria (Pseudomonas), Firmicutes (Paenibacillus), Bacteroidetes (Flavobacterium)*	Growth of individual strains	Antimicrobial production; differential growth rates	Competition varied at different incubation temperatures	[29]
Various Antarctic soils	**Antimicrobial producers**: *Actinobacteria (Arthrobacter), Firmicutes (Planococcus), Gammaproteobacteria (Pseudomonas)*; **Affected:** *Firmicutes (Listeria, Staphylococcus, Brocothrix), Gammaproteobacteria (Salmonella, Escherichia, Pseudomonas)*	Growth of individual strains	Antimicrobial production	Producers were Antarctic bacteria, while affected bacteria were food-borne pathogens	[11]
King George Island, Antarctica	**Antimicrobial producers:** *Bacteroidetes (Pedobacter), Gammaproteobacteria (Pseudomonas);* **Affected:** *Gammaproteobacteria (Salmonella, Escherichia, Klebsiella, Enterobacter, Vibrio), Firmicutes (Bacillus)*	Growth of individual strains	Antimicrobial production	Producers were Antarctic bacteria, while affected bacteria were food-borne pathogens	[30]
Tundra wetland soil, Ural, Russia	Methanogens and homoacetogenic *Firmicutes* (*Acetobacterium*)	H_2_ consumption	Differential H_2_ affinity	Competition was modeled based on changing H_2_ affinities at various temperatures; some strains isolated from pond and fen sediments	[31]
*In situ*					
Unvegetated contaminated soil in Alert, Nunavut, Canada	*Alpha-, Beta-, Gammaproteobacteria, Actinobacteria*	Assimilation of added monoammonium phosphate	Differential nutrient uptake	Alphaproteobacteria most effectively assimilated added nutrients	[32]
*Soil microcosms*					
Lowland soil, Devon Island, Nunavut, Canada	Archaeal and bacterial nitrifiers, fungal and bacterial denitrifiers	N_2_O production, nitrate availability, biomass of microbial domains	Differential nutrient uptake	Effects varied with temperature	[33]

Microbial activity has been demonstrated at temperatures as low as −15 °C [34], but the effects of microbial competition on biogeochemical flux are likely to be most substantial over the summer, as warmer temperatures lead to higher overall activity. Nevertheless, extreme cold can restrict polar microorganisms to small brine pockets at subzero temperatures [35], which may lead to enhanced competition between microorganisms that remain active over winter, as they have reduced opportunities to separate spatially. Competition in polar soils will occur passively due to differential adaptations to soil and environmental conditions, but also actively, as a number of polar soil microorganisms are known to produce inhibitory concentrations of antimicrobial compounds [11,29,30]. The outcome of a change in the abundance of specific groups may substantially affect biogeochemical processes when scaled to entire polar landscapes.

## 2. Factors Influencing the Relative Success of Polar Microorganisms

Adaptations to certain environmental factors, such as extreme cold, will be widespread in polar microbial communities. As in lower latitude soils, taxa will vary in their competitiveness under different environmental conditions, and in the presence of specific co-occurring taxa. This variation will play a large role in determining microbial community composition in polar soils, and will ultimately influence the functional potential of these communities. Assuming that species are not equally efficient at performing a given function (e.g., substrate degradation), small shifts in environmental factors may have substantial effects on the growth and productivity or key microorganisms, as they are limited by competitors that are better adapted to the environment. Below we discuss some of the factors that are known to affect microbial community composition in polar soils.

### 2.1. Environmental Factors

At least at a coarse taxonomic scale, the soil environment appears to be more influential than geography in determining the relative abundance of microorganisms. Recent studies have shown that a main determinant of bacterial composition in polar soils is pH [26,36,37,38,39]. Among bacteria, the major shift due to pH is the increasing abundance of *Acidobacteria* below pH 6 [26,39]. Some studies have observed no effect of pH on bacterial communities in polar soils [40,41], but most of the soils examined had a pH of ~6 or higher. Soil pH has also been shown to correlate somewhat with fungal community composition in polar soils [42,43], while an extensive study of culturable fungal abundance across Antarctic soils showed that fungal abundance declines significantly with increasing pH [44], suggesting an increased importance of bacterial communities.

Other main determinants of community composition include organic matter [41,45], and nitrogen concentration [45,46,47]. Arctic soils with low organic matter content (<10% dry weight of soil) have been shown to favor *Actinobacteria*, while soils with higher organic matter (>10% dry weight of soil) favored an abundance of *Proteobacteria* [41]. High concentrations of nitrogen have generally promoted *Actinobacteria* and *Firmicutes* across biomes [46], as well as *Alpha* and *Gammaproteobacteria* in some Arctic tundra soils [47], although the effect of nitrogen on community composition may largely depend on existing soil organic matter [41]. Fungi in both the Arctic and Antarctic appear to be influenced by C:N ratios [42,43,44], although nutrient additions have sometimes failed to impact certain fungal groups [48,49].

Water content has also been correlated with the bacterial and archaeal community structure of polar soils [41,50,51], although it may have a greater impact on fungi and other microeukaryotes [44,52,53]. Oxygen has also been suggested as an important influence on community structure [54], although this has not been thoroughly tested independent of other factors. Oxygen concentrations will be closely related to soil water saturation, and will determine the dominant forms of metabolism that can occur in soil. Competition may play an important role at anoxic interfaces, when both aerobic and anaerobic forms of metabolism can occur. Other influences on community composition that have been identified from polar environments include phosphorous [43], micronutrients such as potassium and calcium [50], salinity [55], UV radiation [56], and soil particle size [40].

Seasonally changing temperatures will also affect the relative abundance of microorganisms. Two main types of microorganisms remain active in cold environments, and these are the stenopsychrophiles (those that do not grow well or at all at high temperatures (>20 °C)) and eurypsychrophiles (those that have wide temperature growth ranges and may grow optimally at high temperatures) [57,58]. Shifting incubation temperatures from 4 °C to 18 °C was shown to affect the growth rate of different Arctic bacterial isolates differently and ultimately influenced the outcome of competition between them [29]. Similarly, growth temperature has been shown to affect the outcome of competition between cold-adapted marine microbial strains [59,60]. Potential biomass and growth rate can also be decoupled in cold-adapted microbes [61,62]. For instance, psychrophilic bacteria and yeast developed a higher overall biomass at 1 °C than at 20 °C, even though growth rates were highest at 20 °C incubation, while the biomass of mesophiles was highest at 20 °C [62].

### 2.2. Biotic Interactions

The abundance of higher organisms tends to decrease with increasing latitude [63], and this may alter the biotic relationships in polar soils. It has been suggested that the simplified trophic structures of Antarctic soils may lead to an increased importance of abiotic factors in determining community composition and biomass [64], yet reduced complexity at higher trophic levels may lead to communities that are dominated more strongly by microbial processes. Although decreased microbial functional and taxonomic diversity has been observed in higher latitude Antarctic soils [37], it is known that highly diverse microbial communities exist at lower latitudes of the Antarctic [37,65], and throughout the Arctic [26,27]. The best-studied interactions are those that occur between co-occurring microorganisms, and between microorganisms and plants, although other polar soil inhabitants such as viruses and bacterivores are known to exert important top-down controls on the biomass and composition of microbial populations [66,67].

Mechanisms that are involved in intermicrobial cooperation and antagonism at lower latitudes have also been identified in polar and/or subpolar soils. For instance, active quorum sensing genes have been identified in a soil from subarctic Alaska [68]. Chemotaxis is an important strategy to competitively position consumers near nutrients, carbon or to evade toxic chemicals, and while little is known about its importance in cold regions [69], it has been identified in an Arctic *Pseudomonas* isolate [70]. As mentioned earlier, various polar microorganisms are known to produce antimicrobial compounds [11,29,30], while antibiotic resistance genes have even been identified from Arctic permafrost cores [71]. It is unknown how frequently horizontal gene transfer occurs, but a number of mobile elements have been identified from Antarctic soils, with evidence of past transfer events [72,73].

Plant and microbial communities also interact in a variety of ways, where mycorrhizal fungi are the most directly influenced due to their symbiotic relationships with plant root systems. The composition of root-associated fungal communities in the high Arctic has been shown to vary by plant species [43] and successional stage [74], while interactions between plant species and nutrient availability also influence fungal abundance [75]. Interactions between plant species are important as well, as the removal of one shrub species led to decreased ectomycorrhizal colonization of another [48]. Mycorrhizae have even been shown to facilitate carbon transfer between individual *Betula nana* plants in the Arctic tundra, increasing the ability of this plant to compete with neighboring species [76], but also presumably increasing the suitable habitat for its fungal symbionts. Bacterial and archaeal communities have also been influenced by the composition of plant communities in the Arctic [77], although sequencing of various plant assemblages in the Antarctic showed little influence of plant type on bacterial composition [65].

## 3. Important Microbial Functions Potentially Affected by Competition in Polar Soils

Functional redundancy is no longer assumed to be widespread in microbial communities and increasing the relative or absolute abundance of specific taxa is likely required to optimize productivity [19,78]. In mixed communities, it is often not the most productive members that dominate, as relative abundance is determined by adaptations to the abiotic and biotic components of the environment. A number of important biogeochemical processes are microbially-mediated in polar regions, and there is evidence that these processes are limited by constraints on key microbial taxa.

### 3.1. Greenhouse Gas Flux

One of the greatest concerns associated with the warming of polar regions is a potential increase in greenhouse gas production by soil microorganisms, which will further accelerate climate change [79]. The main reasons for this projection are that previously frozen organic matter will become available for degradation, and that microbial activity, previously restricted by low temperatures, is expected to increase. The production and mitigation of gases such as methane and nitrous oxide is restricted to specific microbial groups, so inevitably the factors that control the abundance and activity of these groups will have a major impact on future gas fluxes. While many active microorganisms release CO_2_, the rate and extent of this process will also vary with the abundance and activity of specific key groups.

The abundance and composition of methanogenic and methanotrophic microbial communities have received substantial research attention, particularly in Arctic soils. Huge methane deposits exist in permafrost [80], and even warming to −3 °C and −6 °C has led to methane emissions from permafrost cores [81]. The influence of competition on methanotrophic communities has not been specifically investigated in polar regions, but a simulated disturbance in rice paddy soil showed that as methanotrophic communities reinhabited the underpopulated soil environment, type II methanotrophs dominated due to their more rapid growth rates, thus reducing methanotrophic diversity and evenness [82]. In response to this shift, methane uptake rates more than doubled, and the authors suggest that under natural conditions, methanotroph activity is constrained by competition. Stable isotope probing of high Arctic methanotrophs showed that type I methanotrophs represented the main active community, and methane oxidation was enhanced by amendment with nitrate mineral salts [83], suggesting that this group may be limited by nutrient competition under natural conditions.

In contrast, methanogens appear to be limited mainly be competition for H_2_. Incubations of methanogenic and homoacetogenic strains isolated from Arctic soil and sediment were conducted at varying temperatures and concentrations of H_2_, and modeling of these relationships demonstrated that methanogens would sometimes be outcompeted by homoacetogens at low temperatures and high partial pressures of H_2_ [31]. Depending on the composition of nutrients present in soil, methanogens may have difficulty gaining access to H_2_. By manipulating nutrient concentrations, it was observed in an anoxic rice paddy soil that nitrate, iron, and sulfate reducers were all more successful in H_2_ acquisition than methanogens when H_2_ was limiting [84]. The amount of methane production per methanogenic cell was shown to vary by several orders of magnitude in different subglacial Arctic and Antarctic environments [85], which demonstrates that reducing constraints on these populations could lead to large increases in methane production.

Nitrous oxide (N_2_O) is another important greenhouse gas, with a warming potential 300 times that of CO_2_ [86]. Although N_2_O is frequently the result of incomplete denitrification, nitrifiers can also release N_2_O as a byproduct of nitrification and/or incomplete nitrifier denitrification [87]. Interestingly, nitrifier release of N_2_O has been shown to be the primary source of N_2_O emitted from soils of Devon Island in the high Canadian Arctic [33]. This process appears to be mainly regulated by intermicrobial competition. Denitrifier activity was not enhanced, even following nitrate addition in water-saturated soils, but the inhibition of fungi led to large N_2_O release by denitrifiers, without a subsequent decrease in nitrifier N_2_O production [33]. This suggests that fungi and denitrifiers compete for nitrate, and that this competition mitigates N_2_O release in the Arctic.

Although many organisms produce CO_2_ as a byproduct of activity, competition between microorganisms can limit the amount that is produced by each, relative to the same organisms in isolation [16]. CO_2_ output is also closely linked with the breakdown of soil organic matter, which is discussed in the following section.

### 3.2. Biodegradation

The decomposition of carbon compounds in soil is a key component of the carbon cycle, and is a precursor to the release of carbon-based greenhouse gases. The decomposition of soil organic matter occurs primarily as a result of microbial activity, and catabolic pathways for extracting energy and carbon from complex hydrocarbon substrates are widespread across microbial taxa. Although all soil microbial groups require some form of carbon substrate, they vary in their rate of carbon substrate use, meaning that the promotion or suppression of specific groups will affect rates of organic matter degradation in polar soils. This may apply equally to the degradation of naturally occurring organic matter, and of contaminating hydrocarbons. For instance, across 71 soils from various ecosystems, *Acidobacteria* were negatively correlated with carbon mineralization, while *Bacteroidetes* and *Betaproteobacteria* were positively correlated with this process [88]. *Betaproteobacteria* were also positively correlated with the degradation of diesel across Arctic soils, but were not always promoted following its addition [41].

The potential for decomposition of natural carbon stores is especially large in the Arctic, where nearly half of the world’s below ground carbon may be contained [89]. Similar genes involved in transforming complex organic matter were identified from various microbial groups in metagenomes and metatranscriptomes from high Arctic peat [90], suggesting that competition for substrates is likely to occur. Although certain microorganisms may specialize in the use of different carbon compounds, competition may still occur for other limiting nutrients and space, resulting in the reduced growth of at least one population. This has been shown with bacterial and fungal populations from lower latitude soils [91]. Without explicit microbial competition studies for polar soils, it is often difficult to separate environmental constraints on activity from effects of community structure and activity. The uptake of added carbon in soils from three representative tundra environments was essentially equal, while subsequent release of methane and CO_2_ varied substantially [92]. It is unclear whether other metabolic routes would be available, as these soils varied widely in water content and likely in oxygen availability.

Competition should similarly be expected to influence the degradation of certain contaminants in polar soils, especially compounds that resemble soil organic matter such as petroleum hydrocarbons. Many microorganisms in polar soils have evolved metabolic pathways to exploit petroleum hydrocarbons as sources of carbon and energy [93,94]. Despite a widespread ability to catabolize these molecules, petroleum-metabolizing bacteria differ in both rate and extent of hydrocarbon degradation [95,96,97]. This suggests that the most efficient hydrocarbon degraders may not be promoted naturally, which does appear to be the case, as soil parameters such as organic matter determine which bacteria dominated diesel-contaminated Arctic soils [41]. Nutrient amendments that are applied generally to soil to stimulate the activity of hydrocarbon degraders may actually promote suboptimal hydrocarbon-degrading communities if specific taxa make better use of these nutrients. Following the addition of monoammonium phosphate to contaminated high Arctic soils, the *Alphaproteobacteria* more efficiently assimilated added nitrogen than did the other major active groups [32], although other groups such as the *Gammaproteobacteria* have been associated with efficient remediation at this site [37,98].

The reduction or modification of microbial competition may also represent a biotechnological opportunity for the treatment of contaminated polar soils. In macroecological systems, the loss of key predators has led to reduced constraints on herbivore populations, which have subsequently depleted available vegetation [99]. In the context of bioremediation, this is a desirable outcome, and in fact the fumigation of soils contaminated with 2,4-dichlorophenoxyacetic acid to reduce native microbial populations led to much higher contaminant reduction by introduced strains [22]. Similarly, the inhibition of certain portions of a microbial community in a diesel-contaminated high Arctic soil led to increased degradation [12], suggesting that natural competitive networks may limit bioremediation efficiency.

### 3.3. Plant Productivity

It is not only competition within the microbial community that can affect ecosystem productivity. Plants are the main source of primary biosynthetic material in terrestrial ecosystems, and are a major global carbon pool [100]. Many microorganisms form symbiotic relationships with plants, and it is thought that over 85% of plant nitrogen may be supplied by fungi in Arctic tundra [101]. Nevertheless, antagonistic relationships between microorganisms and plants are known to occur in polar soils. Of these, the best studied involve competition for limiting nutrients such as nitrogen and phosphorus. Reduced nutrient uptake by plants inevitably limits potential biomass, and may seriously impact primary productivity in polar soils.

In Arctic terrestrial environments, the microbial biomass holds a disproportionate amount of the available nutrients when compared with lower latitude ecosystems [102]. In high latitude soils, plant biomass is also likely to be limited by extreme environmental factors such as freezing temperatures, and long-term snow cover. Nutrient additions can promote plant growth several-fold and this effect is more pronounced in the absence of soil microorganisms [102]. When nutrients do become available, they are often quickly assimilated by microorganisms. Irrespective of the form of nitrogen added, 40–50 times more nitrogen ended up in microbial biomass than in plants, in highly acidic (pH 4.6) and mildly acidic (pH 6.4) Arctic tundra soil [103]. This indicates that effective competition for nitrogen may be widespread across microbial taxa, as distinct microbial communities should be expected to exist in these soils [26]. This competitive relationship has also been shown explicitly, as soil sterilization led to increased nitrogen and phosphorus uptake by an Arctic graminoid (*Festuca vivipara*), and increased plant growth, while glucose addition stimulated microbial nutrient uptake, leading to lower plant nutrient acquisition [104].

Plants appear to be more competitive in nutrient acquisition over time, as the ultimate distribution of nutrient pools depends on temporal trends such as the turnover of microbial biomass and plant roots [105]. Although microbial biomass declined in the absence of plants in an Arctic salt marsh, added nitrogen was retained for longer than it was when plants were present [106], suggesting that plants retain nitrogen following microbial turnover. Clemmensen *et al.* [107] also demonstrated that in Arctic soils dominated by *Betula nana*, microbial communities were initially far more efficient at acquiring added nitrogen, but that plants obtained a larger share after less than a month of incubation. Changing seasonal conditions are also likely to affect competitive relationships. In the Arctic, microorganisms appear to accumulate nutrients over the winter [106], but may lose nutrients to plant roots each spring [106,108]. Although plant competition for nutrients is generally considered only for inorganic nutrient sources, plants in polar soils have also been shown to use amino acids and peptides [109,110,111]. In fact, the Antarctic hair grass (*Deschampsia antarctica*) competes successfully with microbial populations for amino acids and peptides, and assimilates peptides much more efficiently than other nitrogen sources [109].

### 3.4. Nutrient Cycling

Although plant-microbe competition for nutrients has been better studied in polar regions, intermicrobial competition may also play an important role in determining the size and composition of nutrient pools. The ability to efficiently acquire limiting nutrients is essential to microbial growth and activity. In addition, certain nutrients will be oxidized or reduced as by-products or end products of metabolic pathways. The combination of nutrient-acquiring and -transforming activities by polar soil microorganisms will determine the size of nutrient pools that are maintained in soils, and that are available to higher trophic levels.

Nitrogen is especially likely to be the subject of widespread competition, as nitrogen availability often limits biomass growth in terrestrial environments [112,113]. The relative abundance of different nitrogen forms will determine which microorganisms will be involved in this competition, as many microorganisms are known to preferentially assimilate NH_4_^+^ over NO_3_^−^, while some are entirely unable to assimilate NO_3_^−^ [114,115,116]. In Arctic tundra soils, ectomycorrhizal fungi were shown to select nitrogen sources other than NO_3_^−^ while effectively sequestering other nitrogen in their mycelia, which may have affected nitrogen selection and use by co-occurring microbes [107]. Similarly, L-alanine and its peptides were equally mineralized by three distinct Antarctic soil microbial communities, while D-alanine was mineralized to different extents and at different rates by each [117], showing that the form of available nitrogen will likely impact which microorganisms are able to remain active in specific soils.

Certain microbial groups are known to be important in nitrogen uptake, and may limit the activity of competitors. Inhibition of fungi in an Arctic tundra soil led to large increases in available NO_3_^−^ [33], while *Alphaproteobacteria* assimilated between 2 and 10 times more added nitrogen than other major active groups in a hydrocarbon-contaminated Arctic soil [32]. While it has been previously suggested that the addition of nitrogen will favour the growth of specific copiotrophic organisms [46,118], it appears that at least in hydrocarbon-contaminated Arctic soils, nitrogen-based fertilizer enhances the competitive advantage of different taxa, depending on soil properties [41]. Competition for nitrogen as both an energy and biosynthetic source may also limit the activity of nitrogen-limited microorganisms. It has been suggested that transformations such as denitrification, which has been observed in hydrocarbon-contaminated Antarctic soils, may limit the nitrogen available to hydrocarbon-degrading taxa [119] as has been observed at lower latitudes [120].

Polar soil microorganisms are also likely to compete for other macronutrients such as phosphorus and sulfur, as well as a variety of micronutrients. A better understanding of the active and potential metabolic routes in polar soils is required in order to speculate on what role such competition might play in affecting important biogeochemical processes.

## 4. The Effects of Environmental Change on Competition

Human activities are causing unprecedented change in the previously isolated polar regions, and a large part of this change is due to rapid climate warming. Much research has been devoted to the effects of warming on polar terrestri al ecosystems, but potential shifts in biogeochemistry are difficult to predict since so many factors are likely to be affected. Although microorganisms are projected to better adapt to this change than other organisms due to their wide physiological range and rapid turnover rate [121], the resulting communities may be substantially changed. The physiology of individual microorganisms will be directly affected by warming, while changes in plant communities and/or soil parameters will likely favor different microbial communities. How these factors will combine to alter competitive relationships between microorganisms in polar soils is unknown, but this will affect the productivity of functions ranging from methane emission to nutrient cycling (Figure 1).

Although some functional redundancy probably exists within natural soil microbial communities, previous disturbances that have altered community composition have frequently shifted microbially-mediated ecosystem processes [122].

Since many of the microorganisms inhabiting seasonally-thawed polar soils are psychrotolerant rather than psychrophilic, increasing temperature should be expected to increase the potential metabolism of many microbial taxa. How this translates into community productivity will depend greatly on competitive interactions. Increased temperature was shown to substantially increase antagonism between many bacterial isolates from Arctic soils, possibly due to increased production of antimicrobials, or shifts in relative growth rates [29]. Long-term warming manipulations in the Arctic led to changes in both bacterial and fungal populations, with increased species evenness among fungi, and decreased evenness among bacteria [5]. Warming manipulations in Antarctic soils led to a more generalist microbial population, as a large decrease in functional richness did not coincide with a decrease in taxonomic richness, suggesting that more species may have been competing to process the same substrates [6]. Such changes may be short-lived, as communities should eventually adapt to new ecological equilibria. Specialization can also rapidly evolve in mixed communities [123], and this divergence may lessen competitive constraints, leading to more rapid resource use. 

A major indirect effect of climate change on microbial communities will arise from changes in plant communities. In the Arctic, the abundance of mycorrhizal plants declines towards the north [124], but climate warming will increase the northward expansion of these plants, increasing bacterial-fungal interactions. Following glacier retreat in the high Arctic, the diversity of ectomycorrhizal fungi increased with increasing plant succession [74]. Warming has also resulted in increased plant success in competing for nutrients with microorganisms in both the Arctic [125] and Antarctic [109]. Interestingly, microorganisms may also better compete with each other by shaping these changing plant communities, and promoting species that favor their growth. Belowground transfer of carbon between *Betula nana* plants in the Arctic was increasingly mediated by fungi with increasing temperature, and helped to establish the dominance of this species [76]. How such changes will affect microbial community productivity in the long-term remains to be seen. Following a 16-year warming experiment in the high Arctic, many changes were observed in the plant communities, while few changes were noted in microbial community structure, or the release of greenhouse gases [126]. This points to a need to understand whether changes in microbial interactions and function following environmental change are transient, or a component of a new community dynamic.

## 5. Studying Competition in Natural Communities

To date, most studies that have examined microbial competition have involved combining a few target species in culture. A key challenge in determining competition in natural communities is that it is difficult to isolate the interactions of specific taxonomic groups. Broad-scale analyses of microbial co-occurrence patterns can establish which taxa are likely to interact frequently, as well as those that are negatively correlated [127]. Many extensive microbial community datasets are now available from polar soils (e.g., [6,26,41]), and meta-analyses may enable prediction of which taxa interact antagonistically. In addition, future studies combining metatranscriptomics and metagenomics will be able to determine whether gene:transcript ratios are equivalent across taxa capable of performing the same function. The advent of high-throughput SIP-proteomic technologies will allow comparisons between transcript and protein abundance [128]. Such studies will help in determining whether the most productive taxa are dominant in particular soils.

Finally, direct manipulation of the abundance of specific taxa within soil may lead to a better understanding of the interactions between key microbial groups. Chloroform fumigation and antibiotic addition can alter microbial diversity and composition in soils, and the resulting effect on activity can then be measured [12,22,129]. In addition, the suppression of specific activities may help in quantifying the contributions of metabolic pathways to bioremediation. This has been used previously to determine the effects of nitrification [130,131,132], nitrogen assimilation [133], denitrification [132,134,135], and sulfate reduction [136] on the nutrient dynamics in soils and sediments. In the future, more specific gene inactivation may also be possible, as RNA external guided sequences have been used in culture to inhibit the expression of targeted mRNA sequences [137], and may eventually be adapted for use in natural environments. Such innovative approaches will be necessary to enhance our understanding of competition in natural microbial communities to include the complex network of interactions that undoubtedly occur.

## 6. Conclusions

Although the importance of microbial interspecies interactions is well recognized, such dynamics have been difficult to assess on a wide scale in natural communities. Certain processes depend upon synergistic interactions, but the niches occupied by particular taxa are often reduced by the growth and activities of co-occurring species that require the same resources and/or space. A characterization of microbial competition in polar soils is desirable for several reasons:

1. Polar soils contain large stores of organic material and nutrients. The extent to which microbial competition can limit rates of decomposition and nutrient cycling will affect climate change predictions and future management plans.
2. By purposefully altering the soil environment, microbial competition may be either increased or reduced, possibly opening biotechnological opportunities such as enhanced bioremediation.
3. Microbial composition and activity also affect the activity and growth of other organisms such as plants, and vice versa. Competition between these groups is also likely to affect the composition and functioning of each.

Future studies that correlate genomic and functional information will help to identify microbial groups that are key to high productivity across polar soils, while manipulation of these communities may reveal some of the constraints that are placed on function due to the coexistence of antagonistic species.

## References

[1] Connell J.H. (1961). The influence of interspecific competition and other factors on the distribution of the barnacle *Chthamalus stellatus*. Ecology.

[2] Huisman J., Weissing F.J. (1999). Biodiversity of plankton by species oscillations and chaos. Nature.

[3] Van Nes E.H., Scheffer M. (2004). Large species shifts triggered by small forces. Am. Nat..

[4] Beninca E., Huisman J., Heerkloss R., Johnk K.D., Branco P., van Nes E.H., Scheffer M., Ellner S.P. (2008). Chaos in a long-term experiment with a plankton community. Nature.

[5] Deslippe J.R., Hartmann M., Simard S.W., Mohn W.W. (2012). Long-term warming alters the composition of arctic soil microbial communities. FEMS Microbiol. Ecol..

[6] Yergeau E., Bokhorst S., Kang S., Zhou J.Z., Greer C.W., Aerts R., Kowalchuk G.A. (2012). Shifts in soil microorganisms in response to warming are consistent across a range of antarctic environments. ISME J..

[7] Barrett L.G., Bell T., Dwyer G., Bergelson J. (2011). Cheating, trade-offs and the evolution of aggressiveness in a natural pathogen population. Ecol. Lett..

[8] Kreth J., Merritt J., Shi W.Y., Qi F.X. (2005). Competition and coexistence between *Streptococcus mutans* and *Streptococcus sanguinis* in the dental biofilm. J. Bacteriol..

[9] Lopez-Garcia S.L., Vazquez T.E.E., Favelukes G., Lodeiro A.R. (2002). Rhizobial position as a main determinant in the problem of competition for nodulation in soybean. Environ. Microbiol..

[10] van Elsas J.D., Chiurazzi M., Mallon C.A., Elhottova D., Kristufek V., Salles J.F. (2012). Microbial diversity determines the invasion of soil by a bacterial pathogen. Proc. Natl. Acad. Sci. USA.

[11] O'Brien A., Sharp R., Russell N.J., Roller S. (2004). Antarctic bacteria inhibit growth of food-borne microorganisms at low temperatures. FEMS Microbiol. Ecol..

[12] Bell T.H., Yergeau E., Juck D., Whyte L.G., Greer C.W. (2013). Alteration of microbial community structure affects diesel degradation in an arctic soil. FEMS Microbiol. Ecol..

[13] Bullock J.M., Pywell R.F., Burke M.J.W., Walker K.J. (2001). Restoration of biodiversity enhances agricultural production. Ecol. Lett..

[14] Doherty J.M., Callaway J.C., Zedler J.B. (2011). Diversity-function relationships changed in a long-term restoration experiment. Ecol. Appl..

[15] Fargione J., Tilman D., Dybzinski R., Lambers J.H., Clark C., Harpole W.S., Knops J.M.H., Reich P.B., Loreau M. (2007). From selection to complementarity: Shifts in the causes of biodiversity-productivity relationships in a long-term biodiversity experiment. Proc. Roy. Soc. B.

[16] Foster K.R., Bell T. (2012). Competition, not cooperation, dominates interactions among culturable microbial species. Curr. Biol..

[17] Peter H., Beier S., Bertilsson S., Lindström E.S., Langenheder S., Tranvik L.J. (2011). Function-specific response to depletion of microbial diversity. ISME J..

[18] Salles J.F., Poly F., Schmid B., Le Roux X. (2009). Community niche predicts the functioning of denitrifying bacterial assemblages. Ecology.

[19] Strickland M.S., Lauber C., Fierer N., Bradford M.A. (2009). Testing the functional significance of microbial community composition. Ecology.

[20] Degens B.P. (1998). Decreases in microbial functional diversity do not result in corresponding changes in decomposition under different moisture conditions. Soil Biol. Biochem..

[21] Griffiths B.S., Ritz K., Bardgett R.D., Cook R., Christensen S., Ekelund F., Sørensen S.J., Bååth E., Bloem J., de Ruiter P.C. (2000). Ecosystem response of pasture soil communities to fumigation-induced microbial diversity reductions: An examination of the biodiversity-ecosystem function relationship. Oikos.

[22] Fournier G., Fournier J.C. (1993). Effect of microbial competition on the survival and activity of 2,4-d-degrading *Alcaligenes xylosoxidans* subsp. *Denitrificans* added to soil. Lett. Appl. Microbiol..

[23] Hibbing M.E., Fuqua C., Parsek M.R., Peterson S.B. (2010). Bacterial competition: Surviving and thriving in the microbial jungle. Nat. Rev. Microbiol..

[24] Little A.E.F., Robinson C.J., Peterson S.B., Raffa K.E., Handelsman J. (2008). Rules of engagement: Interspecies interactions that regulate microbial communities. Annu. Rev. Microbiol..

[25] Roesch L.F., Fulthorpe R.R., Riva A., Casella G., Hadwin A.K.M., Kent A.D., Daroub S.H., Camargo F.A.O., Farmerie W.G., Triplett E.W. (2007). Pyrosequencing enumerates and contrasts soil microbial diversity. ISME J..

[26] Chu H.Y., Fierer N., Lauber C.L., Caporaso J.G., Knight R., Grogan P. (2010). Soil bacterial diversity in the arctic is not fundamentally different from that found in other biomes. Environ. Microbiol..

[27] Neufeld J.D., Mohn W.W. (2005). Unexpectedly high bacterial diversity in arctic tundra relative to boreal forest soils, revealed by serial analysis of ribosomal sequence tags. Appl. Environ. Microb..

[28] McMahon S.K., Wallenstein M.D., Schimel J.P. (2011). A cross-seasonal comparison of active and total bacterial community composition in arctic tundra soil using bromodeoxyuridine labeling. Soil Biol. Biochem..

[29] Prasad S., Manasa P., Buddhi S., Singh S.M., Shivaji S. (2011). Antagonistic interaction networks among bacteria from a cold soil environment. FEMS Microbiol. Ecol..

[30] Wong C.M.V.L., Tam H.K., Alias S.A., Gonzalez M., Gonzalez-Rocha G., Dominguez-Yevenes M. (2011). *Pseudomonas* and *pedobacter* isolates from king george island inhibited the growth of foodborne pathogens. Pol. Polar Res..

[31] Kotsyurbenko O.R., Glagolev M.V., Nozhevnikova A.N., Conrad R. (2001). Competition between homoacetogenic bacteria and methanogenic archaea for hydrogen at low temperature. FEMS Microbiol. Ecol..

[32] Bell T.H., Yergeau E., Martineau C., Juck D., Whyte L.G., Greer C.W. (2011). Identification of nitrogen-incorporating bacteria in petroleum-contaminated arctic soils by using [^15^n]DNA-based stable isotope probing and pyrosequencing. Appl. Environ. Microb..

[33] Siciliano S.D., Ma W.K., Ferguson S., Farrell R.E. (2009). Nitrifier dominance of arctic soil nitrous oxide emissions arises due to fungal competition with denitrifiers for nitrate. Soil Biol. Biochem..

[34] Steven B., Niederberger T.D., Bottos E.M., Dyen M.R., Whyte L.G. (2007). Development of a sensitive radiorespiration method for detecting microbial activity at subzero temperatures. J. Microbiol. Methods.

[35] D’Amico S., Collins T., Marx J.C., Feller G., Gerday C. (2006). Psychrophilic microorganisms: Challenges for life. EMBO Rep..

[36] Fierer N., Jackson R.B. (2006). The diversity and biogeography of soil bacterial communities. Proc. Natl. Acad. Sci. USA.

[37] Yergeau E., Schoondermark-Stolk S.A., Brodie E.L., Dejean S., DeSantis T.Z., Goncalves O., Piceno Y.M., Andersen G.L., Kowalchuk G.A. (2009). Environmental microarray analyses of antarctic soil microbial communities. ISME J..

[38] Chong C.W., Pearce D.A., Convey P., Tan I.K.P. (2012). The identification of environmental parameters which could influence soil bacterial community composition on the antarctic peninsula: A statistical approach. Antarct Sci..

[39] Mannisto M.K., Tiirola M., Haggblom M.M. (2007). Bacterial communities in arctic fjelds of finnish lapland are stable but highly ph-dependent. FEMS Microbiol. Ecol..

[40] Ganzert L., Lipski A., Hubberten H.W., Wagner D. (2011). The impact of different soil parameters on the community structure of dominant bacteria from nine different soils located on livingston island, south shetland archipelago, antarctica. FEMS Microbiol. Ecol..

[41] Bell T.H., Yergeau E., Maynard C., Juck D., Whyte L.G., Greer C.W. (2013). Predictable bacterial composition and hydrocarbon degradation in arctic soils following diesel and nutrient disturbance. ISME J..

[42] Dennis P.G., Rushton S.P., Newsham K.K., Lauducina V.A., Ord V.J., Daniell T.J., O'Donnell A.G., Hopkins D.W. (2012). Soil fungal community composition does not alter along a latitudinal gradient through the maritime and sub-antarctic. Fungal Ecol..

[43] Fujimura K.E., Egger K.N. (2012). Host plant and environment influence community assembly of high arctic root-associated fungal communities. Fungal Ecol..

[44] Arenz B.E., Blanchette R.A. (2011). Distribution and abundance of soil fungi in antarctica at sites on the peninsula, ross sea region and mcmurdo dry valleys. Soil Biol. Biochem..

[45] Powell S.M., Bowman J.P., Ferguson S.H., Snape I. (2010). The importance of soil characteristics to the structure of alkane-degrading bacterial communities on sub-antarctic macquarie island. Soil Biol. Biochem..

[46] Ramirez K.S., Craine J.M., Fierer N. (2012). Consistent effects of nitrogen amendments on soil microbial communities and processes across biomes. Glob. Change Biol..

[47] Campbell B.J., Polson S.W., Hanson T.E., Mack M.C., Schuur E.A.G. (2010). The effect of nutrient deposition on bacterial communities in arctic tundra soil. Environ. Microbiol..

[48] Urcelay C., Bret-Harte M.S., Diaz S., Chapin F.S. (2003). Mycorrhizal colonization mediated by species interactions in arctic tundra. Oecologia.

[49] Robinson C.H., Saunders P.W., Madan N.J., Pryce-Miller E.J., Pentecost A. (2004). Does nitrogen deposition affect soil microfungal diversity and soil n and p dynamics in a high arctic ecosystem?. Glob. Change Biol..

[50] Stomeo F., Makhalanyane T.P., Valverde A., Pointing S.B., Stevens M.I., Cary C.S., Tuffin M.I., Cowan D.A. (2012). Abiotic factors influence microbial diversity in permanently cold soil horizons of a maritime-associated antarctic dry valley. FEMS Microbiol. Ecol..

[51] Hoj L., Rusten M., Haugen L.E., Olsen R.A., Torsvik V.L. (2006). Effects of water regime on archaeal community composition in arctic soils. Environ. Microbiol..

[52] Fell J.W., Scorzetti G., Connell L., Craig S. (2006). Biodiversity of micro-eukaryotes in antarctic dry valley soils with <5% soil moisture. Soil Biol. Biochem..

[53] Bridge P.D., Newsham K.K. (2009). Soil fungal community composition at mars oasis, a southern maritime antarctic site, assessed by pcr amplification and cloning. Fungal Ecol..

[54] Liebner S., Harder J., Wagner D. (2008). Bacterial diversity and community structure in polygonal tundra soils from samoylov island, lena delta, siberia. Int. Microbiol..

[55] Aislabie J.M., Jordan S., Barker G.M. (2008). Relation between soil classification and bacterial diversity in soils of the ross sea region, antarctica. Geoderma.

[56] Tosi S., Onofri S., Brusoni M., Zucconi L., Vishniac H. (2005). Response of antarctic soil fungal assemblages to experimental warming and reduction of uv radiation. Polar Biol..

[57] Feller G., Gerday C. (2003). Psychrophilic enzymes: Hot topics in cold adaptation. Nat. Rev. Microbiol..

[58] Cavicchioli R. (2006). Cold-adapted archaea. Nat. Rev. Microbiol..

[59] Harder W., Veldkamp H. (1971). Competition of marine psychrophilic bacteria at low temperatures. Antonie Van Leeuwenhoek.

[60] Nedwell D.B., Rutter M. (1994). Influence of temperature on growth rate and competition between two psychrotolerant antarctic bacteria: Low temperature diminishes affinity for substrate uptake. Appl. Environ. Microb..

[61] Knoblauch C., Jorgensen B.B. (1999). Effect of temperature on sulphate reduction, growth rate and growth yield in five psychrophilic sulphate-reducing bacteria from arctic sediments. Environ. Microbiol..

[62] Margesin R. (2009). Effect of temperature on growth parameters of psychrophilic bacteria and yeasts. Extremophiles.

[63] Hillebrand H. (2004). On the generality of the latitudinal diversity gradient. Am. Nat..

[64] Hogg I.D., Cary S.C., Convey P., Newsham K.K., O’Donnell A.G., Adams B.J., Aislabie J., Frati F., Stevens M.I., Wall D.H. (2006). Biotic interactions in antarctic terrestrial ecosystems: Are they a factor?. Soil Biol. Biochem..

[65] Teixeira L.C.R.S., Peixoto R.S., Cury J.C., Sul W.J., Pellizari V.H., Tiedje J., Rosado A.S. (2010). Bacterial diversity in rhizosphere soil from antarctic vascular plants of admiralty bay, maritime antarctica. ISME J..

[66] Allen B., Willner D., Oechel W.C., Lipson D. (2010). Top-down control of microbial activity and biomass in an arctic soil ecosystem. Environ. Microbiol..

[67] Newsham K.K., Rolf J., Pearce D.A., Strachan R.J. (2004). Differing preferences of antarctic soil nematodes for microbial prey. Eur. J. Soil Biol..

[68] Williamson L.L., Borlee B.R., Schloss P.D., Guan C.H., Allen H.K., Handelsman J. (2005). Intracellular screen to identify metagenomic clones that induce or inhibit a quorum-sensing biosensor. Appl. Environ. Microb..

[69] Deming J.W. (2002). Psychrophiles and polar regions. Curr. Opin. Microbiol..

[70] Lifshitz R., Kloepper J.W., Scher F.M., Tipping E.M., Laliberte M. (1986). Nitrogen-fixing pseudomonads isolated from roots of plants grown in the canadian high arctic. Appl. Environ. Microb..

[71] D’Costa V.M., King C.E., Kalan L., Morar M., Sung W.W.L., Schwarz C., Froese D., Zazula G., Calmels F., Debruyne R. (2011). Antibiotic resistance is ancient. Nature.

[72] Ma Y.F., Wang L., Shao Z.Z. (2006). *Pseudomonas*, the dominant polycyclic aromatic hydrocarbon-degrading bacteria isolated from antarctic soils and the role of large plasmids in horizontal gene transfer. Environ. Microbiol..

[73] Martinez-Rosales C., Fullana N., Musto H., Castro-Sowinski S. (2012). Antarctic DNA moving forward: Genomic plasticity and biotechnological potential. FEMS Microbiol. Lett..

[74] Fujiyoshi M., Yoshitake S., Watanabe K., Murota K., Tsuchiya Y., Uchida M., Nakatsubo T. (2011). Successional changes in ectomycorrhizal fungi associated with the polar willow *salix polaris* in a deglaciated area in the high arctic, svalbard. Polar Biol..

[75] Sundqvist M.K., Giesler R., Graae B.J., Wallander H., Fogelberg E., Wardle D.A. (2011). Interactive effects of vegetation type and elevation on aboveground and belowground properties in a subarctic tundra. Oikos.

[76] Deslippe J.R., Simard S.W. (2011). Below-ground carbon transfer among *betula nana* may increase with warming in arctic tundra. New Phytol..

[77] Chu H.Y., Neufeld J.D., Walker V.K., Grogan P. (2011). The influence of vegetation type on the dominant soil bacteria, archaea, and fungi in a low arctic tundra landscape. Soil Sci. Soc. Am. J..

[78] Reed H.E., Martiny J.B.H. (2007). Testing the functional significance of microbial composition in natural communities. FEMS Microbiol. Ecol..

[79] Singh B.K., Bardgett R.D., Smith P., Reay D.S. (2010). Microorganisms and climate change: Terrestrial feedbacks and mitigation options. Nat. Rev. Microbiol..

[80] Wagner D., Liebner S., Margesin R. (2009). Global warming and carbon dynamics in permafrost soils: Methane production and oxidation. Permafrost Soils.

[81] Wagner D., Gattinger A., Embacher A., Pfeiffer E.M., Schloter M., Lipski A. (2007). Methanogenic activity and biomass in holocene permafrost deposits of the lena delta, siberian arctic and its implication for the global methane budge. Glob. Change Biol..

[82] Ho A., Luke C., Frenzel P. (2011). Recovery of methanotrophs from disturbance: Population dynamics, evenness and functioning. ISME J..

[83] Martineau C., Whyte L.G., Greer C.W. (2010). Stable isotope probing analysis of the diversity and activity of methanotrophic bacteria in soils from the canadian high arctic. Appl. Environ. Microb..

[84] Achtnich C., Bak F., Conrad R. (1995). Competition for electron donors among nitrate reducers, ferric iron reducers, sulfate reducers, and methanogens in anoxic paddy soil. Biol. Fertil. Soils.

[85] Stibal M., Wadham J.L., Lis G.P., Telling J., Pancost R.D., Dubnick A., Sharp M.J., Lawson E.C., Butler C.E.H., Hasan F. (2012). Methanogenic potential of arctic and antarctic subglacial environments with contrasting organic carbon sources. Glob. Change Biol..

[86] IPCC (2007). Climate Change 2007: The Physical Science Basis.

[87] Wrage N., Velthof G.L., van Beusichem M.L., Oenema O. (2001). Role of nitrifier denitrification in the production of nitrous oxide. Soil Biol. Biochem..

[88] Fierer N., Bradford M.A., Jackson R.B. (2007). Toward an ecological classification of soil bacteria. Ecology.

[89] Tarnocai C., Canadell J.G., Schuur E.A.G., Kuhry P., Mazhitova G., Zimov S. (2009). Soil organic carbon pools in the northern circumpolar permafrost region. Glob. Biogeochem. Cycles.

[90] Tveit A., Schwacke R., Svenning M.M., Urich T. (2013). Organic carbon transformations in high-arctic peat soil: Key functions and microorganisms. ISME J..

[91] Meidute S., Demoling F., Bååth E. (2008). Antagonistic and synergistic effects of fungal and bacterial growth in soil after adding different carbon and nitrogen sources. Soil Biol. Biochem..

[92] Zak D.R., Kling G.W. (2006). Microbial community composition and function across an arctic tundra landscape. Ecology.

[93] Greer C.W., Whyte L.G., Niederberger T.D., Timmis K.N. (2010). Microbial communities in hydrocarbon-contaminated temperate, tropical, alpine, and polar soils. Handbook of Hydrocarbon and Lipid Microbiology.

[94] Aislabie J., Saul D.J., Foght J.M. (2006). Bioremediation of hydrocarbon-contaminated polar soils. Extremophiles.

[95] Ciric L., Philp J.C., Whiteley A.S. (2010). Hydrocarbon utilization within a diesel-degrading bacterial consortium. FEMS Microbiol. Lett..

[96] Sorkhoh N.A., Ghannoum M.A., Ibrahim A.S., Stretton R.J., Radwan S.S. (1990). Crude-oil and hydrocarbon-degrading strains of *rhodococcus-rhodochrous* isolated from soil and marine environments in kuwait. Environ. Pollut..

[97] Whyte L.G., Hawari J., Zhou E., Bourbonnière L., Inniss W.E., Greer C.W. (1998). Biodegradation of variable-chain-length alkanes at low temperatures by a psychrotrophic *rhodococcus* sp. Appl. Environ. Microb..

[98] Yergeau E., Sanschagrin S., Beaumier D., Greer C.W. (2012). Metagenomic analysis of the bioremediation of diesel-contaminated canadian high arctic soils. PLoS One.

[99] Beschta R.L., Ripple W.J. (2009). Large predators and trophic cascades in terrestrial ecosystems of the western united states. Biol. Conserv..

[100] Falkowski P., Scholes R.J., Boyle E., Canadell J., Canfield D., Elser J., Gruber N., Hibbard K., Hogberg P., Linder S. (2000). The global carbon cycle: A test of our knowledge of earth as a system. Science.

[101] Hobbie J.E., Hobbie E.A. (2006). ^15^n in symbiotic fungi and plants estimates nitrogen and carbon flux rates in arctic tundra. Ecology.

[102] Jonasson S., Michelsen A., Schmidt I.K. (1999). Coupling of nutrient cycling and carbon dynamics in the arctic, integration of soil microbial and plant processes. Appl. Soil Ecol..

[103] Nordin A., Schmidt I.K., Shaver G.R. (2004). Nitrogen uptake by arctic soil microbes and plants in relation to soil nitrogen supply. Ecology.

[104] Schmidt I.K., Michelsen A., Jonasson S. (1997). Effects of labile soil carbon on nutrient partitioning between an arctic graminoid and microbes. Oecologia.

[105] Hodge A., Robinson D., Fitter A. (2000). Are microorganisms more effective than plants at competing for nitrogen?. Trends Plant Sci..

[106] Buckeridge K.M., Jefferies R.L. (2007). Vegetation loss alters soil nitrogen dynamics in an arctic salt marsh. J. Ecol..

[107] Clemmensen K.E., Sorensen P.L., Michelsen A., Jonasson S., Strom L. (2008). Site-dependent n uptake from n-form mixtures by arctic plants, soil microbes and ectomycorrhizal fungi. Oecologia.

[108] Edwards K.A., McCulloch J., Kershaw G.P., Jefferies R.L. (2006). Soil microbial and nutrient dynamics in a wet arctic sedge meadow in late winter and early spring. Soil Biol. Biochem..

[109] Hill P.W., Farrar J., Roberts P., Farrell M., Grant H., Newsham K.K., Hopkins D.W., Bardgett R.D., Jones D.L. (2011). Vascular plant success in a warming antarctic may be due to efficient nitrogen acquisition. Nat. Clim. Change.

[110] Henry H.A.L., Jefferies R.L. (2003). Plant amino acid uptake, soluble n turnover and microbial n capture in soils of a grazed arctic salt marsh. J. Ecol..

[111] Chapin F.S., Moilanen L., Kielland K. (1993). Preferential use of organic nitrogen for growth by a nonmycorrhizal arctic sedge. Nature.

[112] Vitousek P.M., Howarth R.W. (1991). Nitrogen limitation on land and in the sea: How can it occur. Biogeochemistry.

[113] Vitousek P.M., Aber J.D., Howarth R.W., Likens G.E., Matson P.A., Schindler D.W., Schlesinger W.H., Tilman D. (1997). Human alteration of the global nitrogen cycle: Sources and consequences. Ecol. Appl..

[114] Imsenecki A.A., Popova L.S., Kirillova N.F. (1976). Effect of nitrogen source on growth of *arthrobacter simplex* and its biosynthesis of cholinesterase. Mikrobiologiâ.

[115] Rice C.W., Tiedje J.M. (1989). Regulation of nitrate assimilation by ammonium in soils and in isolated soil microorganisms. Soil Biol. Biochem..

[116] Recous S., Mary B., Faurie G. (1990). Microbial immobilization of ammonium and nitrate in cultivated soils. Soil Biol. Biochem..

[117] Hill P.W., Farrell M., Roberts P., Farrar J., Grant H., Newsham K.K., Hopkins D.W., Bardgett R.D., Jones D.L. (2011). Soil- and enantiomer-specific metabolism of amino acids and their peptides by antarctic soil microorganisms. Soil Biol. Biochem..

[118] Fierer N., Lauber C.L., Ramirez K.S., Zaneveld J., Bradford M.A., Knight R. (2012). Comparative metagenomic, phylogenetic and physiological analyses of soil microbial communities across nitrogen gradients. ISME J..

[119] Powell S.M., Ferguson S.H., Snape I., Siciliano S.D. (2006). Fertilization stimulates anaerobic fuel degradation of antarctic soils by denitrifying microorganisms. Environ. Sci. Technol..

[120] Roy R., Greer C.W. (2000). Hexadecane mineralization and denitrification in two diesel fuel-contaminated soils. FEMS Microbiol. Ecol..

[121] Callaghan T.V., Bjorn L.O., Chernov Y., Chapin T., Christensen T.R., Huntley B., Ims R.A., Johansson M., Jolly D., Jonasson S. (2004). Biodiversity, distributions and adaptations of arctic species in the context of environmental change. AMBIO.

[122] Allison S.D., Martiny J.B.H. (2008). Resistance, resilience, and redundancy in microbial communities. Proc. Natl. Acad. Sci. USA.

[123] Lawrence D., Fiegna F., Behrends V., Bundy J.G., Phillimore A.B., Bell T., Barraclough T.G. (2012). Species interactions alter evolutionary responses to a novel environment. PLoS Biol..

[124] Olsson P.A., Eriksen B.E., Dahlberg A. (2004). Colonization by arbuscular mycorrhizal and fine endophytic fungi in herbaceous vegetation in the canadian high arctic. Can. J. Bot..

[125] Schmidt I.K., Jonasson S., Shaver G.R., Michelsen A., Nordin A. (2002). Mineralization and distribution of nutrients in plants and microbes in four arctic ecosystems: Responses to warming. Plant Soil.

[126] Lamb E.G., Han S., Lanoil B.D., Henry G.H.R., Brummell M.E., Banerjee S., Siciliano S.D. (2011). A high arctic soil ecosystem resists long-term environmental manipulations. Glob. Change Biol..

[127] Barbéran A., Bates S.T., Casamayor E.O., Fierer N. (2012). Using network analysis to explore co-occurrence patterns in soil microbial communities. ISME J..

[128] Pan C.L., Fischer C.R., Hyatt D., Bowen B.P., Hettich R.L., Banfield J.F. (2011). Quantitative tracking of isotope flows in proteomes of microbial communities. Mol. Cell. Proteomics.

[129] Griffiths B.S., Kuan H.L., Ritz K., Glover L.A., McCaig A.E., Fenwick C. (2004). The relationship between microbial community structure and functional stability, tested experimentally in an upland pasture soil. Microb. Ecol..

[130] Deni J., Penninckx M.J. (1999). Nitrification and autotrophic nitrifying bacteria in a hydrocarbon-polluted soil. Appl. Environ. Microb..

[131] Powell S.J., Prosser J.I. (1986). Inhibition of ammonium oxidation by nitrapyrin in soil and liquid culture. Appl. Environ. Microb..

[132] Bremner J.M., McCarty G.W., Yeomans J.C., Chai H.S. (1986). Effects of phosphoroamides on nitrification, denitrification, and mineralization of organic nitrogen in soil. Commun. Soil Sci. Plant.

[133] Myrold D.D., Posavatz N.R. (2007). Potential importance of bacteria and fungi in nitrate assimilation in soil. Soil Biol. Biochem..

[134] Bremner J.M., Yeomans J.C. (1986). Effects of nitrification inhibitors on denitrification of nitrate in soil. Biol. Fertil. Soils.

[135] Yeomans J.C., Bremner J.M. (1986). Effects of urease inhibitors on denitrification in soil. Commun. Soil Sci. Plant.

[136] Winfrey M.R., Ward D.M. (1983). Substrates for sulfate reduction and methane production in intertidal sediments. Appl. Environ. Microb..

[137] Shen N., Ko J.H., Xiao G.P., Wesolowski D., Shan G., Geller B., Izadjoo M., Altman S. (2009). Inactivation of expression of several genes in a variety of bacterial species by egs technology. Proc. Natl. Acad. Sci. USA.

